# Gender Variation in the Shape of Superior Talar Dome: A Cadaver Measurement Based on Chinese Population

**DOI:** 10.1155/2018/6087871

**Published:** 2018-07-04

**Authors:** Da-Hang Zhao, Di-Chao Huang, Gong-Hao Zhang, Jia-Qi Shi, Chen Wang, Xiang Geng, Xu Wang, Xin Ma

**Affiliations:** Department of Orthopedics, Huashan Hospital, Fudan University, Shanghai 200040, China

## Abstract

Understanding the shape of superior talar dome is essential for a better size compatibility between talar component of ankle implant and bone. The purpose of this study was to determine whether there were gender variations in (1) width (TW) and length (TL) of talus, as well as anterior width (DAW), middle width (DMW), posterior width (DPW), and length (DL) of superior talar dome; (2) differences between the DAW, DMW, and DPW; (3) the ratios between these parameters. Fifty-one cadaveric ankle specimens were included. Two observers measured all the specimens using vernier caliper. Intraclass correlation coefficients (ICCs) were used for intraobserver and interobserver reliability analysis and the reliability was thought to be good if the ICC>0.75. A two-tailed unpaired* t*-test or the rank-sum test was used to investigate gender variations. A single-factor ANOVA was utilized to identify the differences between the width of the superior talar dome surface and p value of <0.05 was considered significant. Intraobserver and interobserver reliability were good. Significant gender variations were found, in which TW, TL, DAW, DMW, DPW, and DL of female specimens were much smaller than those of male. The width of talar dome linearly decreased from DAW to DPW; however, the linearly decreased rate from anterior to posterior width was bigger in female. Moreover, significant differences were found in DAW/DPW, DMW/DPW, DL/DAW, DL/DMW, and DL/DPW between male and female. Based on our result, there was no difference in the 2D shape of the whole talus instead gender variation existed in the 2D shape of superior talar dome between male and female. The current 2D data could contribute to figure out more suitable size of talar component for Chinese population and might indicate a gender-specific shape of bone-implant interface, which could reduce the potential bone-component incompatibility when performing ankle replacement using standard component.

## 1. Introduction

The talus, which is the second largest tarsus of the foot, is essential for normal gait mechanics [1, 2]. It is important to do measurement of talar dome when aiming to design more anatomical ankle implant [3]. Research on the three-dimensional (3D) morphology of talar dome contributes to design the morphology of superior articular surface of talar component while research on two-dimensional (2D) shape of talar dome could help us to figure out the shape and size of bone-implant interface of talar component [2–4]. It was revealed that sizes of the current ankle implant designs may differ considerably from real joint dimensions and inappropriate size of prosthesis might result in postoperative complications, e.g., impingement or subsidence [5–7]. One study suggested that the widths of the talar components of HINTEGRA were not completely compatible to the ankles from Korean population [8]. It was presumed that Chinese population may indicate similar result.

Some studies have measured the geometry of the talar dome. It was suggested that anterior, middle, and posterior widths of trochlea were all longer from male than those from female formalin-fixed cadaveric talar specimen [8]. Another study, which based on constructed 3D computer-assisted talar models of Chinese population, suggested similar results [9]. However, a CT study of Caucasian US adult cohort revealed different results, in which a significant gender difference was found in anterior and posterior width except the middle width [10], and the width linearly decreased from anterior to posterior in both male and female [10]. All these results suggest gender differences existed in the length of the mentioned parameters. Meanwhile one study indicated no gender difference was found in the ratio of anterior width and length of talar dome [8]. A published study indicated the anterior-posterior to medial-lateral aspect ratio of femoral condyle was larger in women than men [11]. But it remains unclear whether there are gender differences in the ratio of anterior and posterior width, as well as width and length of talar trochlea which could indicated the potential variation in shape of superior talar dome.

The purpose of the present study was to investigate whether there were gender variations in (1) talus length (TL), talus width (TW), anterior width of the superior talar dome surface (DAW), middle width of the superior talar dome surface (DMW), posterior width of the superior talar dome surface (DPW), and the length of the superior talar dome surface (DL); (2) differences between the width of the superior talar dome surface; and (3) TW/TL, DAW/DMW, DAW/DPW, DMW/DPW, DAW/DL, DMW/DL, and DPW/DL based on fresh frozen cadavers of Chinese population.

## 2. Materials and Methods

### 2.1. Cadaveric Talar Specimens

The fresh frozen cadaveric below-knee lower extremity specimens were obtained from donors without previous trauma, deformity, or degenerative changes. All the specimens were from department of Human Anatomy & Histoembryology, Shanghai Medical College, Fudan University, and were evaluated independently by three of authors (DZ, DH, GZ) with any disagreements in eligibility resolved by consensus discussion. The study was conducted in accordance with the Declaration of Helsinki. The protocol was approved by the Ethics Committee of Huashan Hospital, Fudan University. Fifty-one cadaveric ankle specimens of Han Chinese population, including 33 male and 18 female, 31 right and 20 left, were included in the present study. The mean age at death was 55.04 (26 to 73).

### 2.2. Parameters of Measurements

**Figure 1:**
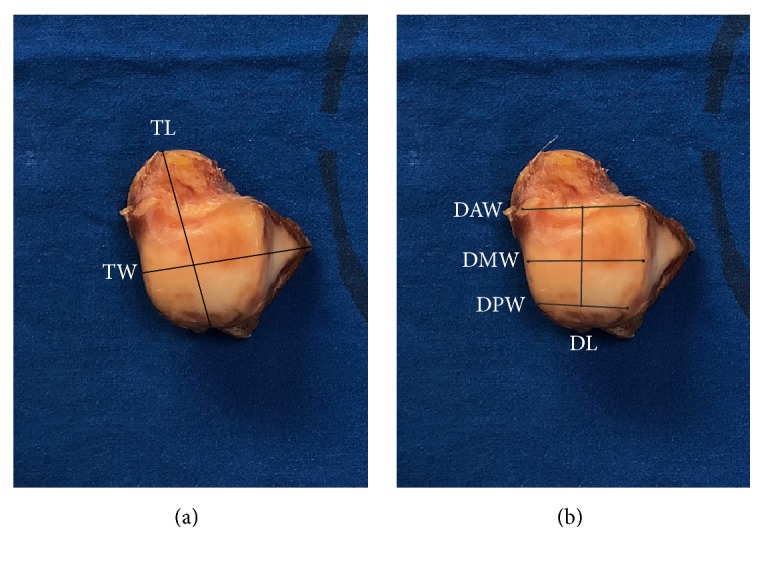
Measured parameters. (a) TL is the distance from the apex of the talar head to the groove of the flexor hallucis longus; TW is the distance from the lateral talar process to the midpoint of the medial talar trochlea. (b) DAW is the distance between the anterior points of the medial and lateral trochlea, DMW is the distance between the apexes of the medial and lateral talar domes, DPW is the distance between the posterior points of the trochlea, and DL is the distance between the midpoint of the DAW and DPW.

In our study, 2 parameters of talus and 4 parameters of trochlea were measured. TW is the width of talus and TL is the length of talus. DAW, DMW, and DPW is anterior, middle, and posterior width of talar dome. DL is the length of talar dome. TL is the distance from the apex of the talar head to the groove of the flexor hallucis longus; TW is the distance from the lateral talar process to the midpoint of the medial talar trochlea (Figure 1(a)). DAW is the distance between the anterior points of the medial and lateral trochlea; DMW is the distance between the apexes of the medial and lateral talar domes and DPW is the distance between the posterior points of the trochlea; and DL is the distance between the midpoint of the DAW and DPW (Figure 1(b)), which were the same as those in previous studies [8–10]. The gender variations analyses were done by mean value of four-time measurements.

### 2.3. Process of Measurement

The cadaver measurement was done by two observers independently using vernier caliper of 0.02 mm accuracy. When a single observer (DZ) did the measurement, a maker pen (with 0.5mm Φ tip) was used to mark the point on the talar dome surface and measure each parameter using carefully without breaking the cartilage. After wiping out the mark by 75% medicine alcohol gauze, a second measurement by the same observer was done. The process of measure was recorded in video (see supplementary video). The second observer (DH) repeated the process independently. All the results were recorded (see Supplementary Material) by another author (GZ).

### 2.4. Statistical Analysis

Intraobserver and interobserver reliability were evaluated in all 50 talus using intraclass correlation coefficients (ICCs). Intraobserver reliability for the two observers was calculated by repeated measurements after wiping out the mark. Interobserver reliability was calculated between the four times separate measurements of 2 observers. The reliability was thought to be good if ICC>0.75, moderate if ICC was 0.50-0.75, and poor if ICC<0.50, using published criteria [12]. A two-tailed unpaired* t*-test was used to investigate gender variations. In the absence of a normal distribution, the rank-sum test was used for comparisons. A single-factor ANOVA was utilized to identify the differences between the width of the superior talar dome surface. When a difference was found, pairwise comparisons were applied. SAS software version 9.2 (SAS Institutes, Cary, North Carolina, USA) was used and p value of <0.05 was considered significant.

## 3. Results

### 3.1. Intraobserver and Interobserver Reliability

The intraobserver and interobserver reliability for TW, TL, DAW, DMW, DPW, and DL were all >0.80 and were regarded as good (Table 1).

### 3.2. Gender Variations in TW, TL, DAW, DMW, DPW, and DL

**Figure 2:**
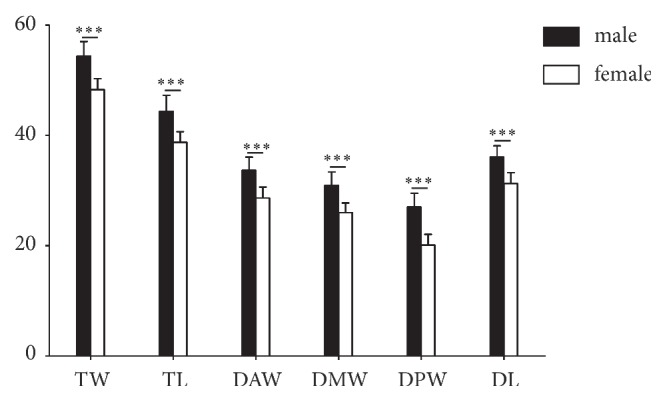
Gender variations in the mean value of TW, TL, DAW, DMW, DPW, and DL with standard deviation (*∗∗∗* means p < 0.001).

The TW, TL, DAW, DMW, DPW, and DL were normally distributed in the entire subjects and for the male and female. The mean values of TW, TL, DAW, DMW, DPW, and DL of the 51 talar specimens were 52.233±3.777mm, 42.391±3.733mm, 31.883±3.329mm, 29.205±3.238mm, 24.608±4.047mm, and 34.361±3.075. The mean values of the 6 parameters were 54.358±2.654 mm, 44.375±2.882 mm, 33.661±2.430 mm, 30.949±2.419 mm, 27.050±2.486 mm, and 36.053±2.091 for male, and 48.337±1.963 mm, 38.753±1.926 mm, 28.624±2.024 mm, 26.007±1.756 mm, 20.130±1.936 mm, and 31.259±1.958 for female, respectively (Table 2). A significant gender difference was found in all the measured parameters that TW (p < 0.001), TL (p < 0.001), DAW (p < 0.001), DMW (p < 0.001), DPW (p < 0.001), and DL (p < 0.001) from talar specimens of female were all smaller than those of male (Table 2 and Figure 2).

### 3.3. Gender Variations in Differences between the Width of the Superior Talar Dome Surface

**Figure 3:**
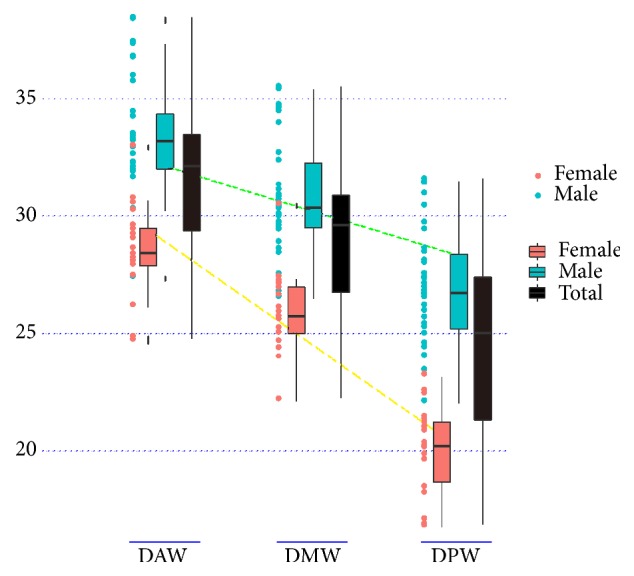
Anterior, middle, and posterior width of superior talar dome surface of the male female and all subjects plotted as points. The averages and standard deviations for male, female, and all subjects were illustrated with neighboring unfilled squares and error bars. It was revealed that the linearly decreased rate from anterior to posterior widths of talar dome was bigger in female (yellow dotted line) than male (green dotted line).

Significant difference was found among DAW, DMW, and DPW by single-factor ANOVA (p < 0.001). Difference pairwise comparisons revealed there were differences between DAW and DMW (p < 0.001), DAW and DPW (p < 0.001), as well as DMW and DPW (p < 0.001) respectively. The average difference from DAW to DMW and DMW to DPW of all the talar specimens were 2.679±0.700 mm and 4.597±1.374 mm. The average decrease from DAW to DMW and DMW to DPW were 2.713±0.707 mm and 3.899±1.004 mm for male and 2.617±0.704 mm and 5.877±0.987 mm for female, respectively (Table 3); however the difference from DMW to DPW had gender variation (p < 0.001). Moreover, the width of the superior talar dome surface linearly decreased from DAW to DPW regardless of specimens of males, females, or those across all subjects (Figure 3).

### 3.4. Gender Variations in the Ratio of TW/TL, DAW/DMW, DAW/DPW, DMW/DPW, DAW/DL, DMW/DL, and DPW/DL

The ratios of TL/TW, DAW/DMW, DAW/DPW, DMW/DPW, DAW/DL, DMW/DL, and DPW/DL were 1.227±0.046, 1.088±0.025, 1.247±0.052, 1.146±0.043, 1.073±0.035, 1.167±0.043, and 1.338±0.070 for male, and 1.248±0.042, 1.101±0.027, 1.427±0.082, 1.297±0.071, 1.093±0.039, 1.203±0.045, and 1.560±0.105 for female (Table 4). Difference between male and female cadaveric talar specimens was found in the ratio of DAW/DPW (p < 0.001), DMW/DPW (p < 0.001), DL/DAW (p = 0.025), DL/DMW (p = 0.005), and DL/DPW (p < 0.001).

## 4. Discussion

Although this study measured the 2D shape of the talar dome in a cadaveric Chinese population, some gender variations were found which included variations on length of TW, TL, DAW, DMW, DPW, and DL and the ratio of DAW/DPW, DMW/DPW, and DL/DPW. To the best of our knowledge, this is the first report in English that investigates gender variations in the size and shape of talar dome through cadaveric measurement on Han Chinese population.

There are some limitations in our study. First, there was no 3D model measurement made and our result could not reflect the morphology of articular surface of talar dome. Second, no data of height and weight of donor was available in this study. However a published study suggested the correlation between height of the subject and talar measurement was poor [5]. Third, intrinsic error may exist when measuring talar specimens by vernier caliper. Although visually identified landmarks might affect reproducibility. We did reliability analysis on our measurement comparing to previous studies of cadaver measurement using vernier caliper [13, 14].

Our results indicated that TW, TL, DAW, DMW, DPW, and DL from talar specimens from male were all bigger than those from female, which were the same as the results from the study based on formalin-fixed specimens from Korean population [8]. The mean values from our study were similar to those from their study. However the values based on fresh frozen talus specimens measurement included thickness of cartilage, which were approximate 1 to 2 mm [15]. However, it was not clear that if the values of these parameters from our study have statistical differences comparing to those from their research even if the results are quite similar. He et al. measured talus models constructed from CT data and also found that leading, middle, and trailing widths were bigger from male [9]. Another CT radiological study, which was based on American population, indicated gender difference was found in both anterior and posterior width except at the middle width [10]. Different methods of measurement might results in our values were a little more than those studies based on CT image. Because it is suggested radiological values were slightly lesser than the anatomical values [16].

Our results suggested the anterior, middle and posterior width of the superior talar dome surface were different from each other, which DAW was longer than DMW as well as DPW. However, our results also suggested the decrease from DMW to DPW was more in specimens of females than those of males, which indicated that the decreased rate from DAW to DPW was bigger in female. On the other side, it was indicated the width of the talar dome linearly decreased from anterior to posterior regardless of male or female (Figure 2), which was consistent with published results [10]. These results indicated the superior talar dome surface was approximately wedged and made it is possible for the assumption that both medial and lateral crests of the talar dome were straight when designing the talar component of ankle implant [17]. The current congruent bicondylar ankle implants comply with this feature which have two straight crests of the talar dome [18].

**Figure 4:**
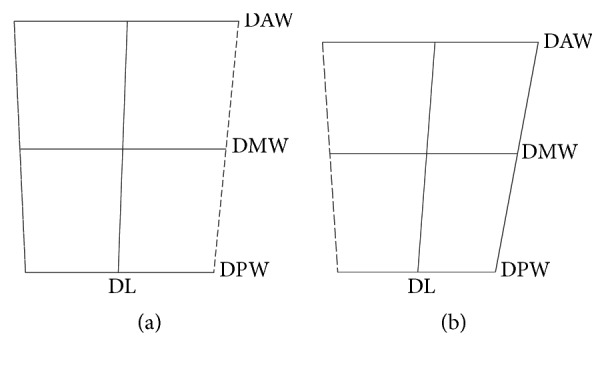
Gender variation in the superior talar dome surface of Chinese population. (a) Illustration of the superior talar dome surface in males. (b) Illustration of the superior talar dome surface in females.

No difference was found in the ratio of TW/TL and DAW/DL which was the same as the results from Eun et al. [8]. No gender variation in the ratio of TW/TL indicated that there was no difference in the 2D shape of the talus between male and female. Instead significant differences between male and female cadaveric specimens were found in the ratio of DAW/DPW, DMW/DPW, DL/DAW, DL/DMW, and DL/DPW suggested that gender variation existed in the 2D shape of superior talar dome, which indicated the diminishing degree of the width of talar dome from anterior to posterior was more in females. One published study revealed the range of frequency distribution for inversion/eversion rotation of the women's ankles was more than those of men's [19]. However, if gender variations in the 2D shape of talar dome that are related to more amount of inversion/eversion motion in females need further investigations. Consider the above-mentioned result that linearly decreased rate from anterior to posterior widths of talar dome was bigger in female (Figure 3). It was presumed that the 2D superior talar dome surface in male was more rectangular and the superior talar dome surface in female was more trapezoidal in Chinese population (Figure 4). The finding was consistent with the published results that inclination angle between medial and lateral crest line of the talar dome was more in female [17]. The significance of the 2D conclusion could help to design a more compatible shape for talar component of ankle prosthesis of male and female. Consequently, if we want to design ankle implant which is more suitable for Chinese population, gender-specific shape of the bone-implant interface might be needed.

Our result of the width and length of talus and talar dome could contribute to figuring out more suitable size of talar component for Chinese population; meanwhile the gender variations in the 2D shape of superior talar dome might indicate a gender-specific shape of bone-implant interface, which could reduce the potential bone-component incompatibility when performing ankle replacement using standard component. Further research is required to investigate the 3D morphology of talar dome in order to design superior articular surface and radius of talar component for Chinese population.

## Figures and Tables

**Table 1:** Intraobserver and interobserver reliability analysis.

Variable	Intraobserver		Interobserver			
	A1 and A2	B1 and B2	A1 and B1	A1 and B2	A2 and B1	A2 and B2
TW	0.964 (0.939 - 0.980)	0.965 (0.940 - 0.980)	0.976 (0.958 - 0.986)	0.970 (0.948 - 0.983)	0.953 (0.918 - 0.973)	0.966 (0.941 - 0.980)
TL	0.975 (0.957 - 0.986)	0.937 (0.893 - 0.964)	0.934 (0.887 - 0.962)	0.982 (0.968 - 0.990)	0.929 (0.879 - 0.959)	0.973 (0.953 - 0.984)
DAW	0.907 (0.843 - 0.946)	0.913 (0.853 - 0.949)	0.936 (0.891 - 0.963)	0.904 (0.838 - 0.944)	0.921 (0.865 - 0.954)	0.915 (0.855 - 0.950)
DMW	0.912 (0.852 - 0.949)	0.850 (0.752 - 0.911)	0.895 (0.822 - 0.938)	0.851 (0.753 - 0.912)	0.920 (0.864 - 0.953)	0.877 (0.794 - 0.928)
DPW	0.948 (0.911 - 0.970)	0.936 (0.892 - 0.963)	0.956 (0.924 - 0.975)	0.952 (0.917 - 0.972)	0.946 (0.906 - 0.969)	0.950 (0.915 - 0.971)
DL	0.913 (0.852 - 0.949)	0.917 (0.859 - 0.952)	0.907 (0.842 - 0.946)	0.914 (0.854 - 0.950)	0.894 (0.821 - 0.938)	0.900 (0.831 - 0.942)

TW: width of the talar; TL: length of the talar; DAW: anterior width of the superior talar dome surface; DMW: middle width of the superior talar dome surface; DPW: posterior width of the superior talar dome surface; DL: length of the superior talar dome surface; A1: the first measurement by observer 1; A2: the second measurement by observer 1; B1: the first measurement by observer 2; B2: the second measurement by observer 2.

**Table 2:** Gender variations in TW, TL, DAW, DMW, DPW, and DL.

Variable	Total	Gender		p value
		Male	Female	
TW	52.233±3.777	54.358±2.654	48.337±1.963	<0.0001
TL	42.391±3.733	44.375±2.882	38.753±1.926	<0.0001
DAW	31.883±3.329	33.661±2.430	28.624±2.024	<0.0001
DMW	29.205±3.238	30.949±2.419	26.007±1.756	<0.0001
DPW	24.608±4.047	27.050±2.486	20.130±1.936	<0.0001
DL	34.361±3.075	36.053±2.091	31.259±1.958	<0.0001

Values represent means ± SD (mm); TW: width of the talar; TL: length of the talar; DAW: anterior width of the superior talar dome surface; DMW: middle width of the superior talar dome surface; DPW: posterior width of the superior talar dome surface; DL: length of the superior talar dome surface.

**Table 3:** Gender variations in the difference between the width of the superior talar dome surface.

Variable	Total	Gender		p value
		Male	Female	
DAW to DMW	2.679±0.700	2.713±0.707	2.617±0.704	0.844
DMW to DPW	4.597±1.374	3.899±1.004	5.877±0.987	<0.0001

Values represent means ± SD (mm); DAW: anterior width of the superior talar dome surface; DMW: middle width of the superior talar dome surface; DPW: posterior width of the superior talar dome surface.

**Table 4:** Gender variations in TL/TW, DAW/DMW, DAW/DPW, DMW/DPW, DL/DAW, DL/DMW, and DL/DPW.

Variable	Gender		p value
	Male	Female	
TL/TW	1.227±0.046	1.248±0.042	0.124
DAW/DMW	1.088±0.025	1.101±0.027	0.079
DAW/DPW	1.247±0.052	1.427±0.082	<0.0001
DMW/DPW	1.146±0.043	1.297±0.071	<0.0001
DL/DAW	1.073±0.035	1.093±0.039	0.025
DL/DMW	1.167±0.043	1.203±0.045	0.005
DL/DPW	1.338±0.070	1.560±0.105	<0.0001

Values represent means ± SD; TW: width of the talar; TL: length of the talar; DAW: anterior width of the superior talar dome surface; DMW: middle width of the superior talar dome surface; DPW: posterior width of the superior talar dome surface; DL: length of the superior talar dome surface.

## Data Availability

The datasets supporting the conclusions of this article are included within the article and its supplementary materials.

## Supplementary Materials

Supplementary Materials The materials of the present study included a supplementary video and a supplementary data, which all are referred to in the manuscript. The supplementary video recorded the measurement process for the talar dome. And the supplementary data is the original measurement results by two observers.
