# Supplementary material for: Gender Variation in the Shape of Superior Talar Dome: A Cadaver Measurement Based on Chinese Population
Source: Biomed Res Int. 2018 Jul 4;2018:6087871. doi: 10.1155/2018/6087871 (PMC6057431; doi:10.1155/2018/6087871)
Supplement: Supplementary Materials — The materials of the present study included a supplementary video and a supplementary data, which all are referred to in the manuscript. The supplementary video recorded the measurement process for the talar dome. And the supplementary data is the original measurement results by two observers. [file 6087871.f1.zip › 6087871.f1.pdf]

Talus	Side	Gender	Age	Observer	TL	TW	DAW	DMW	DPW	DL
1	L	F	56	A1	47.48	36.34	25.92	21.88	16.12	27.66
				A2	48.38	37.02	23.96	22.92	17.18	28.56
				B1	46.46	37.48	25.04	23.06	17.2	26.62
				B2	47.42	38.42	24.18	21.02	18.06	27.6
2	L	F	48	A1	51.74	40.82	31.6	27.94	20.88	32.06
				A2	53.72	43.12	29.48	27.86	21.94	33.16
				B1	53.68	42.04	31.54	26.02	22.04	31.18
				B2	52.76	43.22	30.5	26.98	21.08	30.22
3	L	F	62	A1	47.78	38.02	29.78	25.46	18.22	29.32
				A2	48.74	37.96	28.62	26.5	17.18	31.5
				B1	47.8	36.74	26.52	24.38	19.32	30.48
				B2	46.88	36.04	26.96	23.96	18.28	31.34
4	L	M	71	A1	53.12	40.26	34.4	32.2	27.54	34.04
				A2	54.06	42.18	33.48	31.16	26.58	36.12
				B1	52.96	41.16	32.54	30.14	28.64	35.2
				B2	53.02	40.2	31.46	31.04	26.68	34.16
5	L	M	63	A1	47.82	36.9	27	25.08	23.62	31.94
				A2	48.8	35.76	26.96	26.14	21.7	31.02
				B1	47.76	36.88	28.86	28.06	20.64	33.88
				B2	47.06	36.04	26.92	27.08	22.58	32.98
6	R	M	59	A1	51.1	42.32	33.48	30.38	27.12	37.78
				A2	52.2	43.9	34.56	31.26	29.08	39.96
				B1	50.98	43.02	32.5	29.34	27.12	38.92
				B2	51.06	42.98	33.62	31.4	26.98	37.84
7	L	F	57	A1	46.58	38.68	27.22	25.96	17.06	31.12
				A2	47.66	37.7	28.08	24.04	16.14	32.04
				B1	48.64	36.74	28.73	23.9	18.18	31.08
				B2	47.66	38.8	29.02	24.98	16.08	29.96
8	R	M	68	A1	51.78	42.3	34.94	31.16	27.96	34.8
				A2	51.14	42.74	33.06	30.04	28.86	35.66
				B1	51.8	41.92	32.88	31.14	29.84	34.78
				B2	52.06	42.82	33.02	29.02	28.02	35.6

9	R	F	55	A1	48.94	38.86	24.92	23.96	16.12	30.18
				A2	48.28	39.02	26.1	24.02	18.28	29.04
				B1	48.82	38.18	24.68	23.88	17.24	28.08
				B2	48.06	38.72	23.94	25.84	16.08	27.16
10	R	M	67	A1	52.86	41.74	33.1	31.38	27.38	35.56
				A2	52.22	42.82	32.44	29.16	24.96	34.6
				B1	53.04	41.86	33.32	28.24	25.4	36.68
				B2	52.36	40.98	34.38	30.28	26.26	35.5
11	R	M	60	A1	51.66	42.1	31.24	27.02	25.1	33.94
				A2	51.12	41.76	33.18	28.98	26.14	34.86
				B1	52.46	40.5	32.22	28.94	24.98	35.14
				B2	51.92	41.4	31.3	36.88	25.04	36.1
12	L	M	26	A1	58.7	45.5	33.48	29.96	28.46	35.76
				A2	60.1	44.72	34.52	31.76	27.38	37.44
				B1	58.94	45.62	35.62	31.02	29.42	36.64
				B2	59.08	44.46	33.46	30.1	27.32	37.58
13	L	M	46	A1	52.96	44.42	31.76	28.48	26.84	33.26
				A2	53.08	45.52	33.76	31.6	25.9	34.18
				B1	52.88	43.66	31.84	28.42	27.98	35.4
				B2	53.12	44.7	30.68	29.38	26.02	34.22
14	L	F	56	A1	45.86	37.96	27.02	25.06	18.52	28.66
				A2	45.28	38.78	25.96	23.92	19.6	29.58
				B1	46.34	38.06	26.88	24.1	18.48	28.74
				B2	45.6	38.7	25.08	23.04	17.4	30.64
15	R	M	65	A1	54.9	41.78	36.88	34.94	30.6	40.34
				A2	54.16	40.92	38.96	35.88	31.76	39.18
				B1	53.92	40.8	37.02	34.92	32.88	38.28
				B2	54.54	41.02	36.94	36.04	30.76	39.24
16	R	F	58	A1	45.78	38.94	27.2	25.32	18.88	30.94
				A2	45.1	37.02	29.36	26.26	20.74	31.02
				B1	46.02	39.08	27.24	25.18	19.68	30.86
				B2	45.68	38.1	26.18	24.36	19.3	29.76

17	R	M	53	A1	55.16	47.66	33.78	32.54	28.68	36.52
				A2	55.94	46.86	33.26	31.42	29.84	34.64
				B1	55.78	47.62	34.04	32.3	30.72	35.34
				B2	55	46.7	35.96	33.36	28.6	36.24
18	R	M	63	A1	56.26	48.02	36.78	35.26	29.86	38.82
				A2	56.48	47.94	35.86	34.38	31.94	39.94
				B1	56.8	49.98	34.68	32.12	30.04	37.86
				B2	56.52	47.88	36.74	34.24	32.08	39.56
19	L	M	69	A1	57.26	49.78	35.98	33.66	32.62	39.08
				A2	58.08	48.82	37.06	34.58	30.74	37.16
				B1	57.98	49.74	37.12	35.52	31.66	38.96
				B2	58.06	48.88	36.98	34.74	30.82	39.4
20	R	M	68	A1	59.96	46.94	37.28	35.6	30.38	39.48
				A2	58.06	47.06	36.36	33.56	31.46	38.66
				B1	58.48	46.84	37.42	35.42	30.54	37.62
				B2	60.32	47.78	38.38	34.48	29.48	37.2
21	R	M	64	A1	56.98	46.74	36.86	34.36	29.64	38.38
				A2	56.1	47.86	35.78	33.54	28.16	36.46
				B1	57.88	46.72	37.76	35.48	27.42	36.02
				B2	56.92	45.8	36.92	34.6	28.82	37.54
22	R	F	67	A1	48.08	36.78	28.38	26.72	20.28	33.96
				A2	47.68	35.94	29.7	25.84	19.4	32.04
				B1	47.92	37.02	27.98	24.52	20.38	30.88
				B2	47.26	36.84	28.6	26.74	21.46	31.96
23	R	M	64	A1	52.2	44.78	31	28.34	25.58	34.4
				A2	52.72	43.92	31.98	29.3	24.32	33.32
				B1	53.18	44.88	30.9	27.22	23.84	35.44
				B2	52.02	44.2	32.94	29.7	24.78	34.5
24	R	M	36	A1	55.72	46.72	32.58	30.08	26.88	34.2
				A2	54.76	45.46	33.66	30.62	28.76	33.16
				B1	55.94	47.54	34.7	31.56	27.02	35.26
				B2	55.34	46.2	32.04	29.44	25.94	34.22

25	L	M	48	A1	51.98	40.22	30.14	26.78	24.7	33.44
				A2	52.12	39.98	31.98	27.56	23.74	32.86
				B1	52.46	40.18	30.08	28.7	24.82	33.96
				B2	51.6	41.14	29.1	27.18	22.94	34.8
26	L	M	63	A1	52.48	44.14	31.82	29.2	26.04	33.88
				A2	52.86	43.24	33.78	31.18	24.98	34.9
				B1	52.2	44.3	32	30.26	25.1	35.84
				B2	53.04	43.44	31.6	29.78	24.04	33.76
27	L	M	45	A1	58.98	49.76	37.78	34.94	31.2	41.38
				A2	60.2	48.96	39.66	35.12	30.06	40.8
				B1	58.34	49.04	38.52	33.18	33.16	39.78
				B2	59.2	49.38	37.68	35.6	32.08	40.04
28	L	M	54	A1	53.2	42.8	31.12	28.06	25.68	34.78
				A2	52.98	43.78	32.06	29.88	26.78	35.82
				B1	53.42	43.9	31.62	30.02	26.74	34.3
				B2	52.84	43.32	33.3	31.08	27.8	33.88
29	R	M	66	A1	54.28	45.02	34.08	31.94	26.02	36.08
				A2	54.94	46.78	35.96	30.12	25.98	35.94
				B1	55.04	45.84	34.04	29.46	27.94	38.16
				B2	54.18	44.98	33.8	32.1	28.08	37.02
30	L	M	67	A1	53.78	45.28	31.96	27.98	23.26	34.02
				A2	54.54	44.46	32.2	29.12	24.06	33.9
				B1	53.72	45.58	33.88	29.66	23.98	34.7
				B2	53.2	46.48	31.3	28.94	25.02	34.12
31	R	M	64	A1	53.16	42.58	32.36	29	25.3	35.38
				A2	53.74	41.96	33.42	29.7	26.48	34.26
				B1	53.26	42.78	34.54	28.96	25.6	35.18
				B2	52.9	42.3	32.84	30.16	27.54	36.28
32	R	M	61	A1	53.74	46.48	31.58	29.92	24.12	35.66
				A2	53.1	45.56	33.04	28.98	23.92	34.4
				B1	52.94	45.38	31.94	28.16	25.08	36.5
				B2	53.82	45.02	34.18	31.9	24.68	34.98

33	R	F	59	A1	51.32	42.24	32.08	30.04	21.88	35.66
				A2	50.56	41.36	33.24	31.06	23.76	34.7
				B1	50.1	42.28	32.62	29.94	22.78	35.24
				B2	50.84	41.54	34.22	31.1	24.62	36.68
34	R	F	57	A1	50.14	41.24	32.04	28.86	22.18	35.62
				A2	49.82	42.08	29.96	26.9	22.96	33.54
				B1	49.3	40.96	31.06	27.88	23.98	32.68
				B2	51.08	41.5	29.28	26.12	21.22	33.08
35	R	F	69	A1	47	36.72	27.86	25.88	20.14	28.28
				A2	46.94	36.2	28.74	26.76	22.06	30.46
				B1	47.78	37.54	27.4	25.68	21.02	29.34
				B2	47.08	36.18	29.68	26.28	20.96	30.06
36	R	F	38	A1	50.42	38.3	30.18	26.38	19.26	32.6
				A2	50.88	38.84	28.26	24.42	20.12	30.22
				B1	50.12	39.44	29.2	26.44	21.18	32.32
				B2	49.8	38.6	28.7	25.28	18.96	31.28
37	R	M	45	A1	52.94	42.94	31.76	29.58	25.84	35.7
				A2	52.38	43.76	32.28	28.96	26.76	36.5
				B1	53.02	42.04	31.24	29.9	25.42	35.44
				B2	52.1	41.84	33.3	30.04	24.88	37.38
38	R	M	50	A1	54.12	43.68	33.7	30.74	26.72	36.14
				A2	54.8	44.76	34.36	32.88	27.58	35.08
				B1	53.98	43.88	32.46	31.06	26.28	37.1
				B2	53.46	44.74	33.42	30.76	25.74	35.6
39	L	M	69	A1	55.54	44.74	32.78	29.78	26.68	36.34
				A2	54.32	46.36	33.9	31.46	27.76	35.28
				B1	55.48	44.06	34.74	32.58	25.84	36.62
				B2	54.56	45.28	31.86	29.48	27.1	37.44
40	R	M	73	A1	53.1	45.04	33.16	30.78	27.28	36.6
				A2	53.76	44.72	34.48	31.82	28.36	37.06
				B1	54.72	46.48	33.5	31.2	27.48	36.04
				B2	54.3	45.66	32.6	29.86	26.56	38.1

41	R	F	49	A1	48.82	36.66	28.66	25.28	21.76	32.96
				A2	48	37.28	27.52	26.2	20.82	31.76
				B1	47.94	36.04	29.74	26.68	22.74	30.78
				B2	47.38	36.8	27.08	24.38	19.62	33.88
42	L	F	58	A1	51.06	38.7	29.66	27.48	22.9	33.28
				A2	49.88	38.66	30.58	28.22	22.26	34.32
				B1	49.42	39.6	29.52	27.04	21.46	32.28
				B2	49.04	37.78	28.78	26.4	23.22	33.64
43	L	F	65	A1	45.18	36.56	27.86	24.68	20.72	30.08
				A2	44.34	35.96	28.18	25.76	19.1	31.64
				B1	45.26	37.1	27.28	25.88	19.96	30.52
				B2	45.92	36.04	29.14	26.72	20.88	32.74
44	L	M	66	A1	51.32	41.3	31.82	27.76	24.44	31.96
				A2	52.46	39.98	29.88	26	23.22	33.02
				B1	53.5	41.14	30.04	27.6	25.3	32.1
				B2	52.58	40.04	29.56	25.88	23.4	32.76
45	R	F	69	A1	49.78	40.74	30.82	27.8	20.54	29.86
				A2	50.06	41.74	28.96	26.54	19.26	29.1
				B1	41.78	39.8	29.06	27.26	20.1	30.02
				B2	49.94	40.03	28.2	26.3	21.04	31.1
46	R	M	57	A1	56.06	45.8	37.14	34.06	27.08	37.74
				A2	55.86	46.7	34.06	31.92	29.88	36.56
				B1	55.34	45.66	36.04	32.14	27.14	37.1
				B2	54.94	46.52	35.86	32.82	28.1	38.72
47	R	F	63	A1	49.88	42.16	30.98	27.32	21.78	33.9
				A2	49.12	41.26	29.14	26.04	20.92	32.14
				B1	50.08	41.3	31.08	28.16	21.04	34.06
				B2	49.1	40.22	29.9	27.02	19.84	32.5
48	R	F	56	A1	47.86	38.7	28.58	27.04	20.16	29.72
				A2	48.04	37.38	29.76	26.86	21.16	30.02
				B1	47.9	38.22	28.7	25.02	21.98	31.88
				B2	47.26	39.36	30.84	27.8	22.24	29.34

49	R	M	42	A1	55.9	46.04	33.54	29.88	24.72	34.84
				A2	56.7	46.78	31.26	28.9	22.96	35.12
				B1	55.48	45.58	32.46	28	23.8	34.3
				B2	55.02	47.52	30.5	27.1	22.36	33.26
50	R	M	38	A1	53.16	42.66	34.12	30.78	24.76	36.44
				A2	52.32	43.82	33.68	28.82	25.52	34.78
				B1	53.6	42.08	32.98	29.84	26.7	32.98
				B2	54.06	42.68	33.04	30.6	24.82	34.08
51	L	M	62	A1	59.42	48.56	39.08	34.62	29.84	41.02
				A2	60.52	47.84	38.12	35.44	29.78	39.1
				B1	58.96	47.2	38.74	36.22	28.9	40.08
				B2	60.64	48.58	37.9	35.9	30.48	41.86
